# Supplementary material for: The influence of X chromosome variants on trait neuroticism
Source: Mol Psychiatry. 2019 Mar 6;26(2):483–91. doi: 10.1038/s41380-019-0388-2 (PMC7850965; doi:10.1038/s41380-019-0388-2)
Supplement: Supplementary file 1 — Supplementary Figures [file 41380_2019_388_MOESM1_ESM.docx]

**Supplementary Figure 1. Q-Q plot of X chromosome association results for neuroticism in UK Biobank.**

**
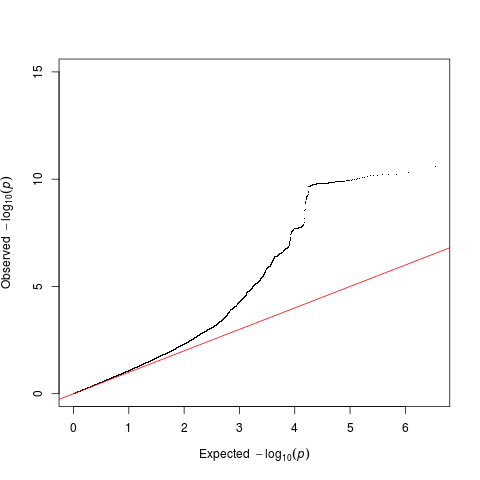
**

**Supplementary Figure 2. Q-Q plot of XY pseudoautosomal association results for neuroticism in UK Biobank.**

**
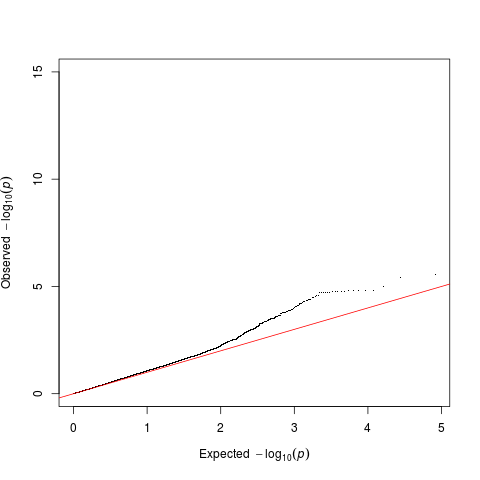
**

**Supplementary Figure 3. X Chromosome association results for neuroticism in UK Biobank women (N = 219,259). XY pseudoautosomal SNPs are in darker blue.**


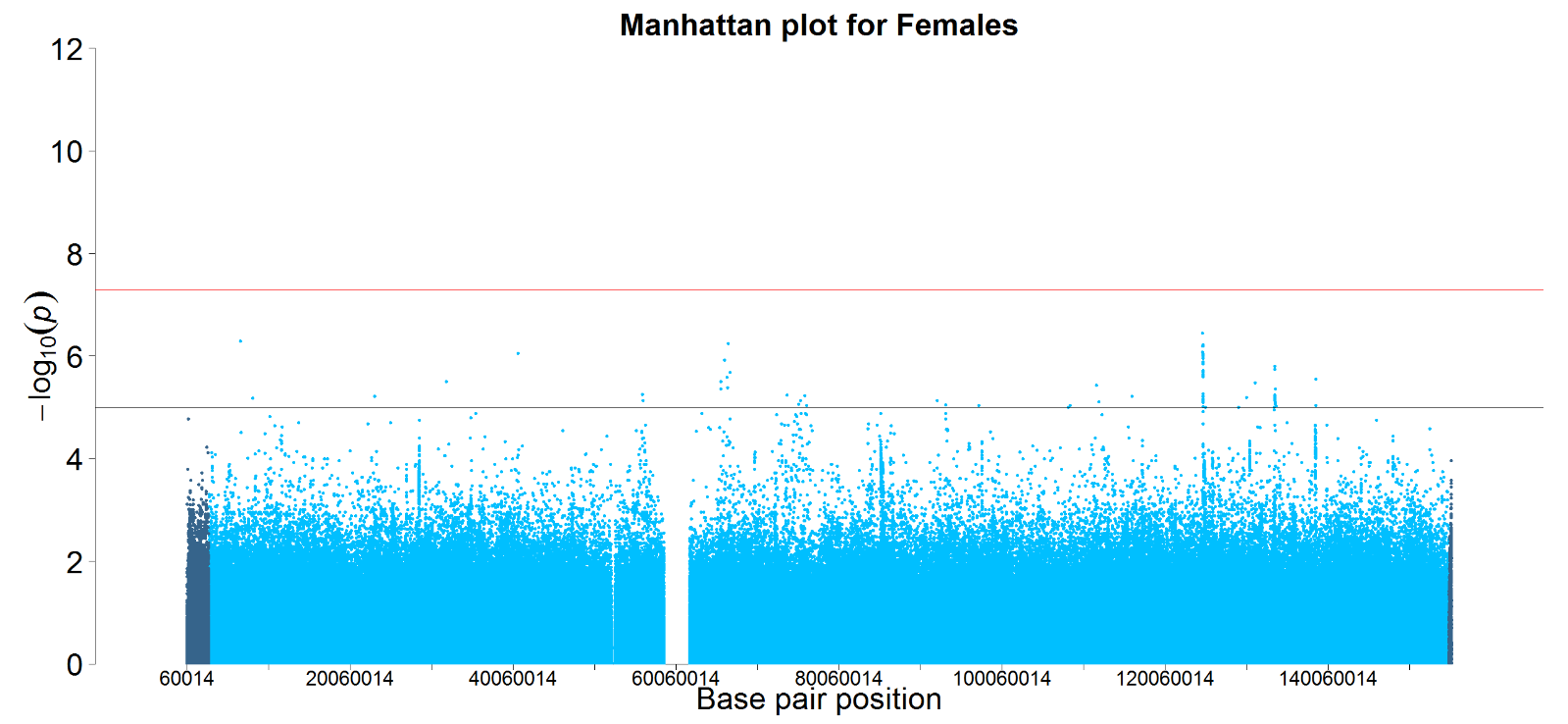


**Supplementary Figure 4. X Chromosome association results for neuroticism in UK Biobank men (N = 186,015). XY pseudoautosomal SNPs are in darker blue.**


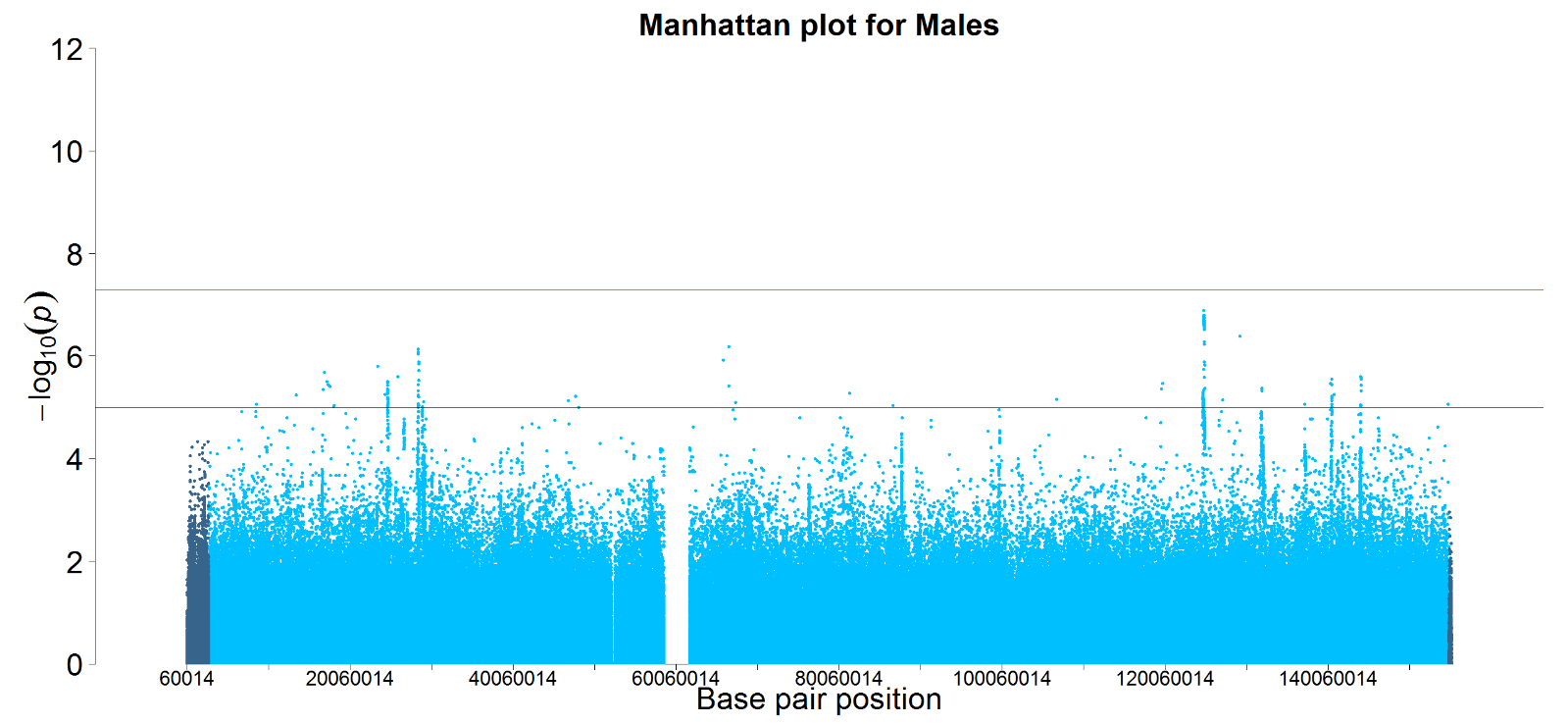


**Supplementary Figure 5. Regional association plot for Locus 1 (Xp21.3)**


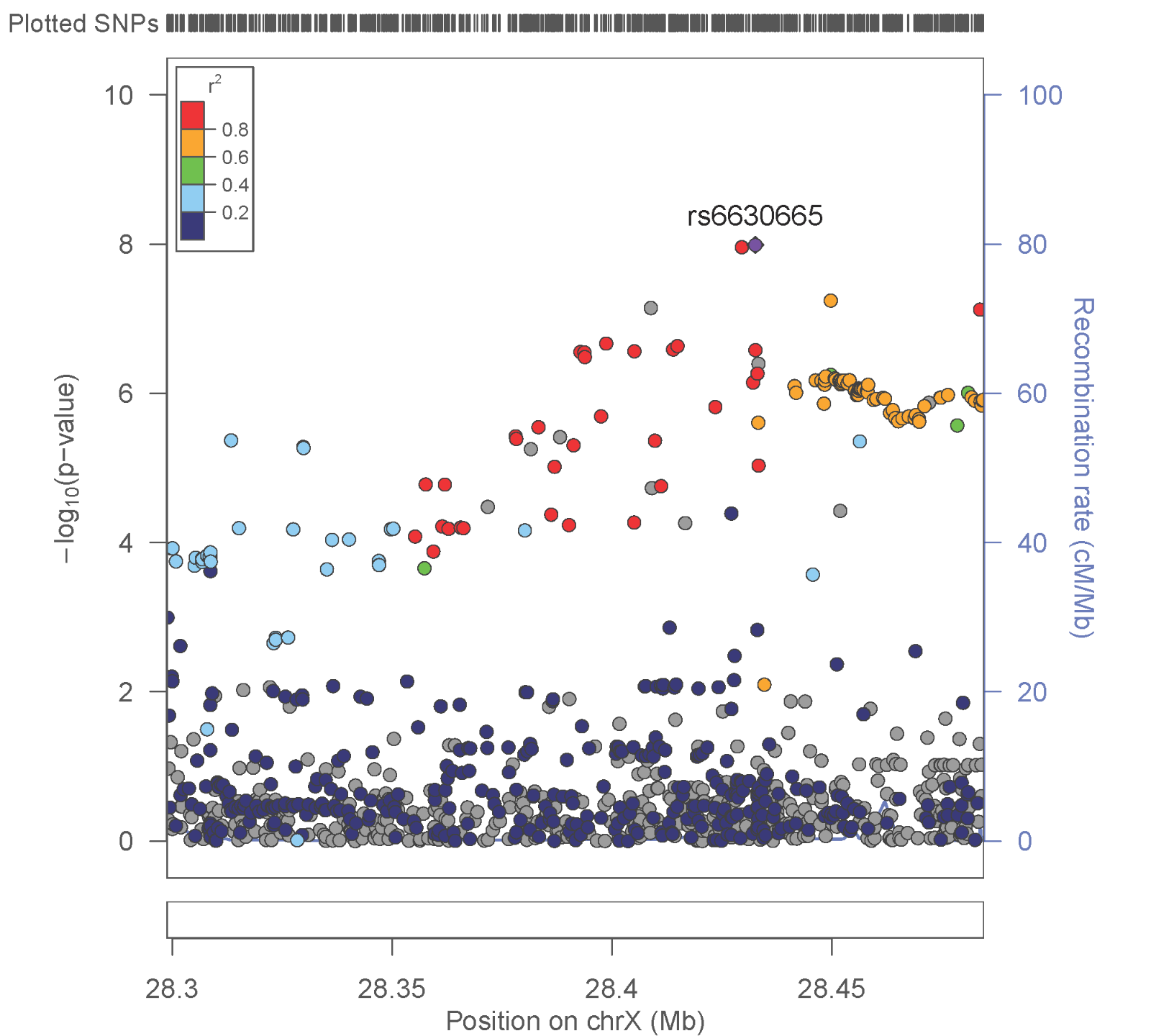


**Supplementary Figure 6. Regional association plot for Locus 2 (Xq25)**


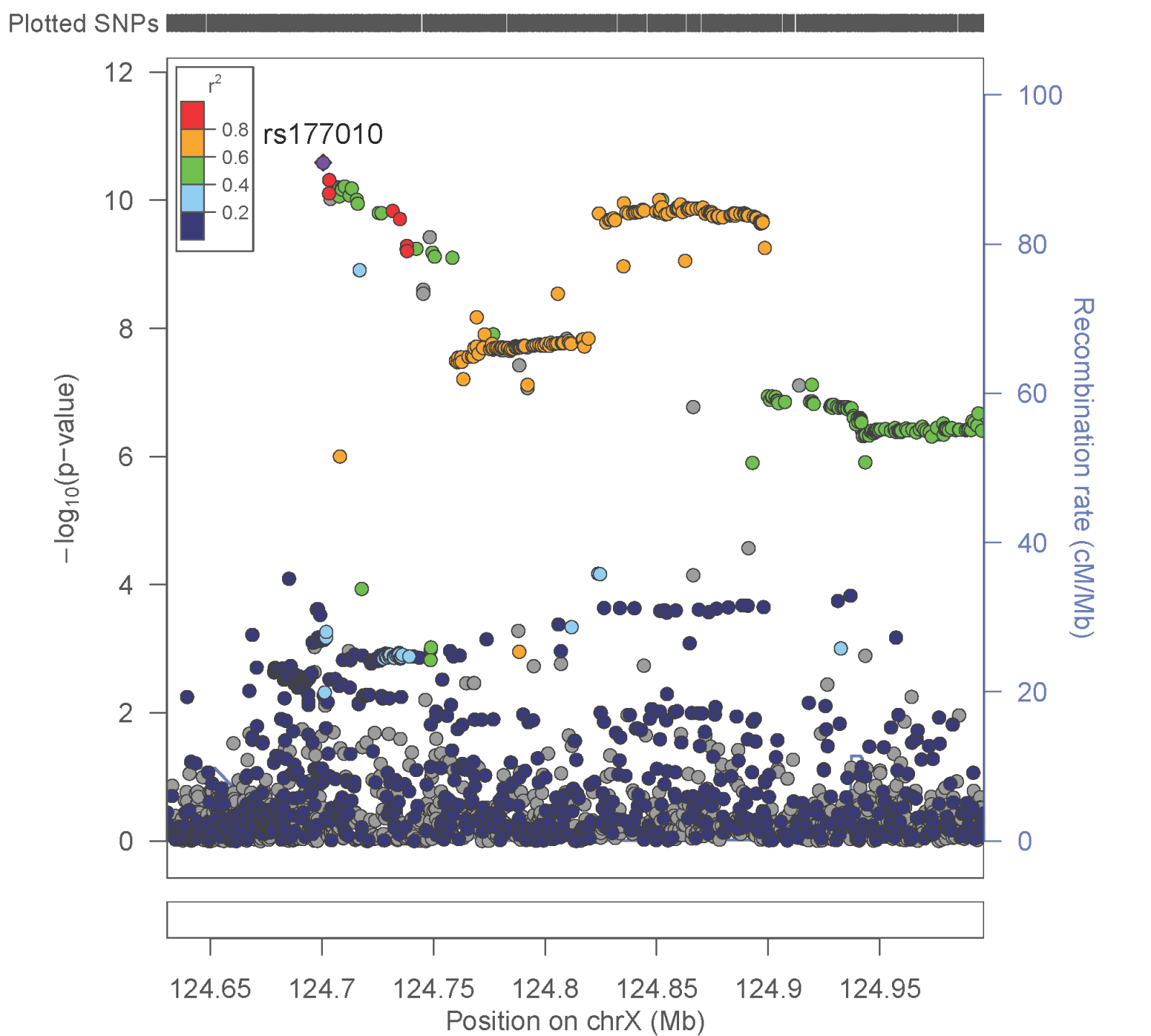


**Supplementary Figure 7. Regional association plot for Locus 3 (Xq26.2)**

**
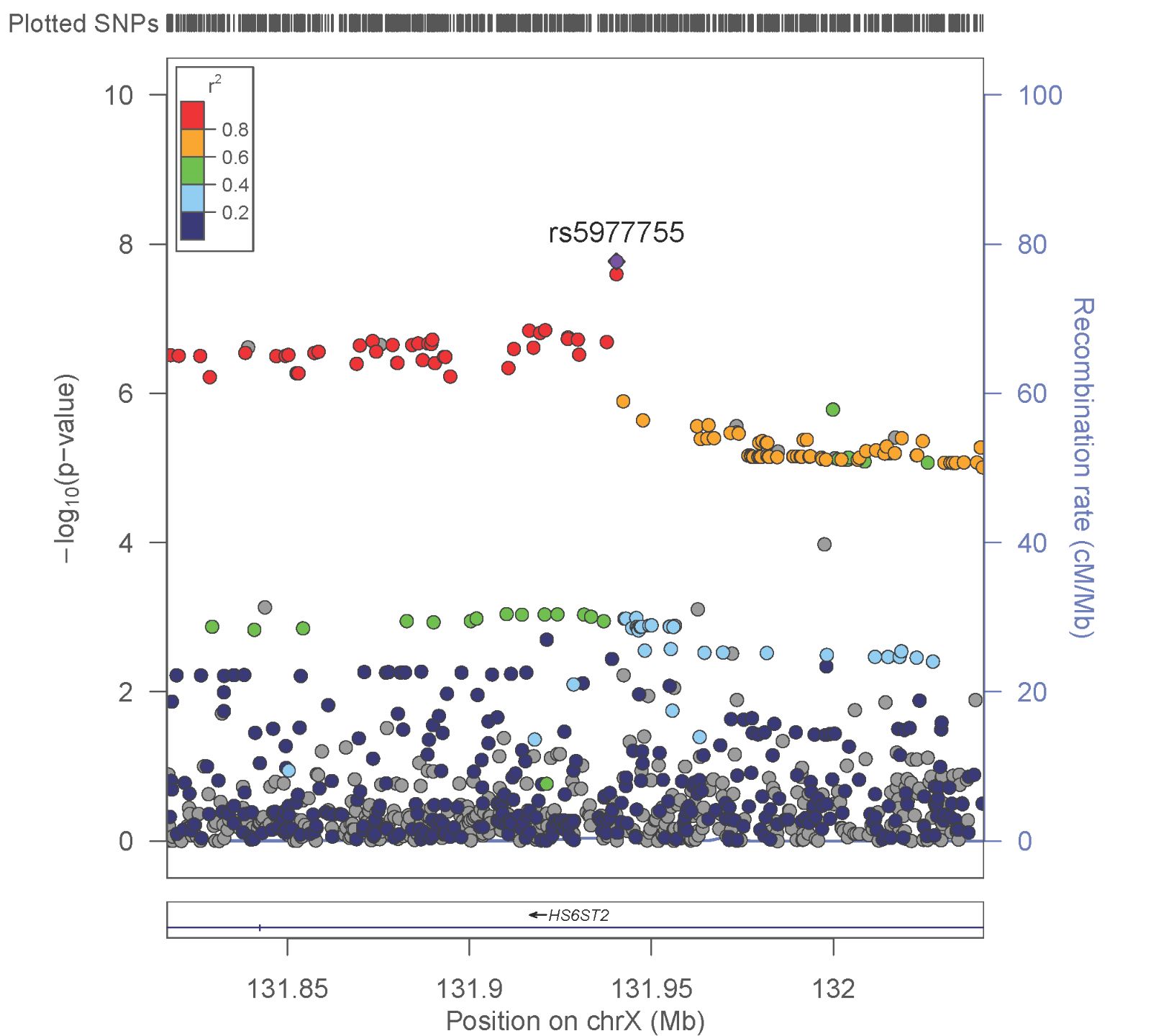
**

**Supplementary Figure 8. Top genes from suggestive X chromosome neuroticism associations: Molecular Function**


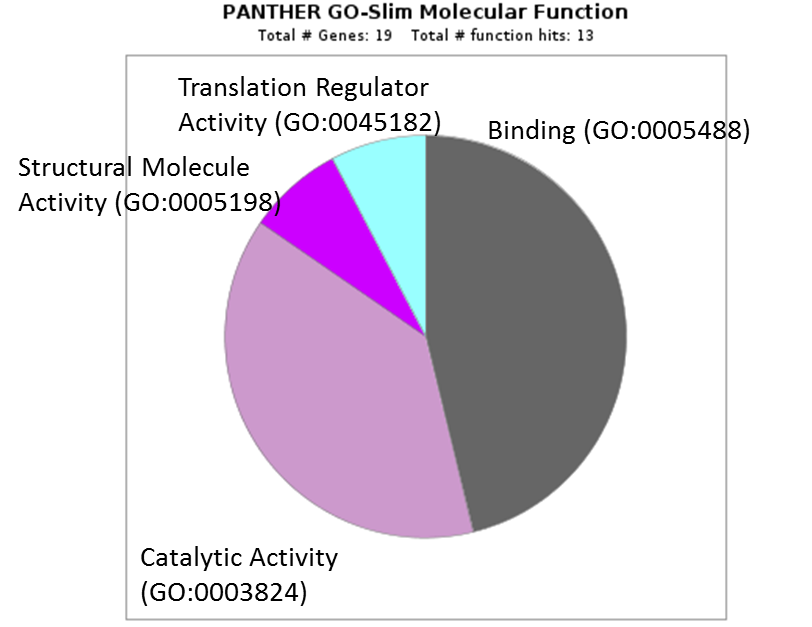


**Supplementary Figure 9. Top genes from suggestive X chromosome neuroticism associations: Cellular Component**


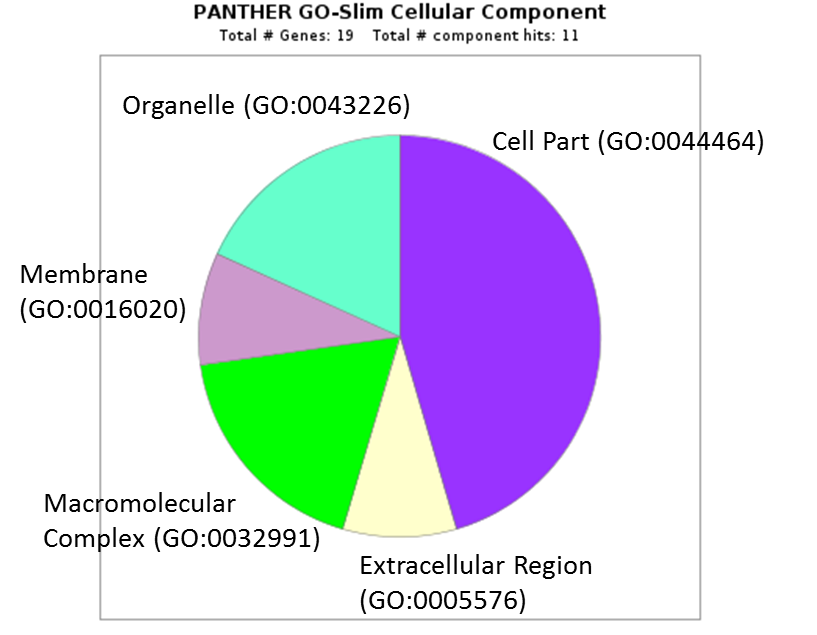


**Supplementary Figure 10. Top genes from suggestive X chromosome neuroticism associations: Biological Process**


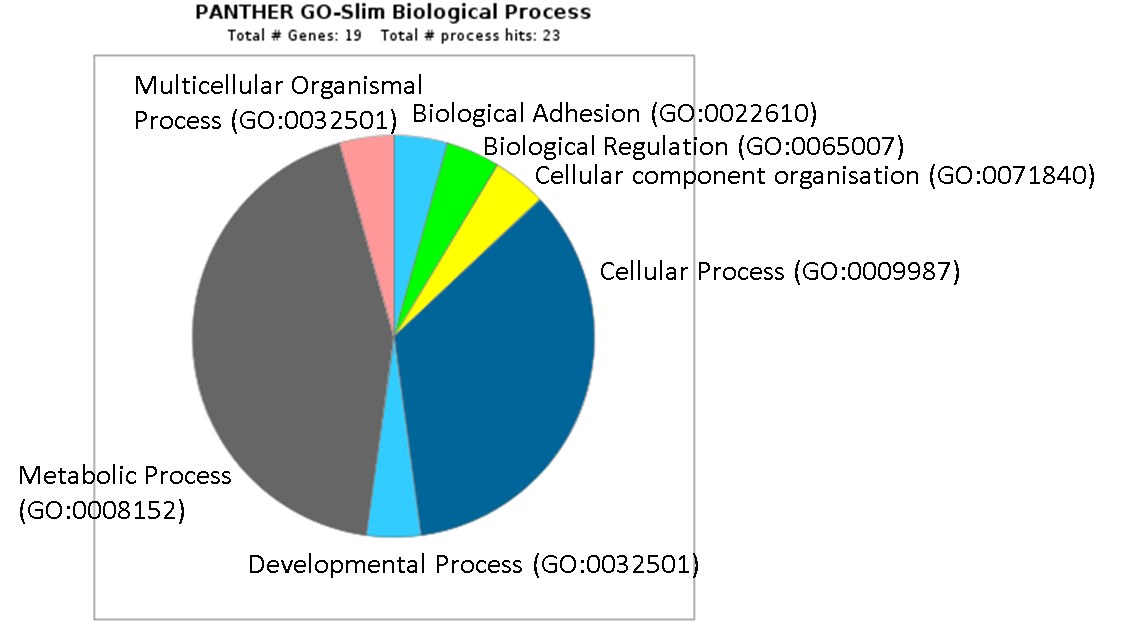


**Supplementary Figure 11. Top genes from suggestive X chromosome neuroticism associations: Protein Class**


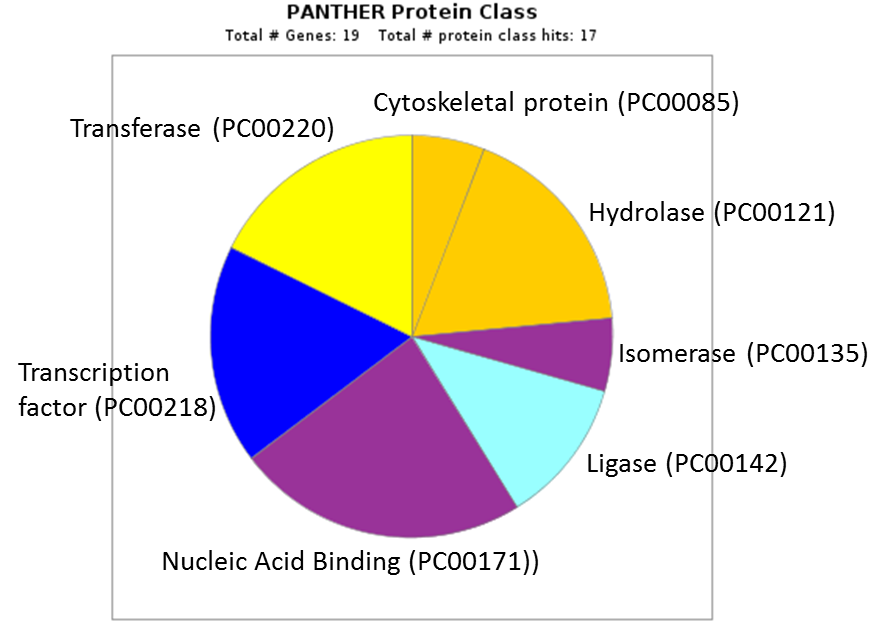


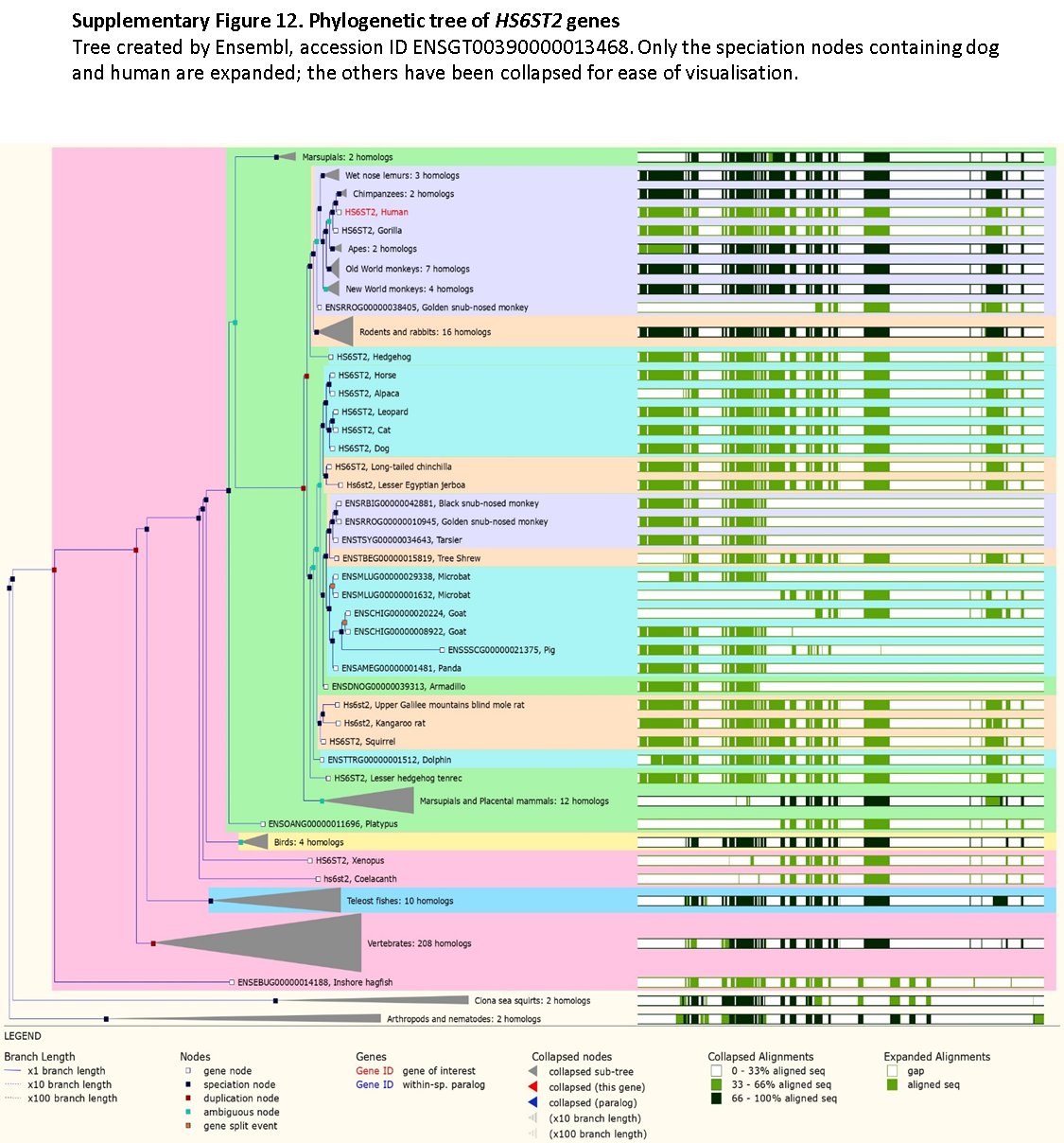


**Supplementary Figure 13. Sequence comparison for HS6ST2 across species.**

Protein sequence from Ensembl, predicted from cDNA sequence. **A.** Alignment of predicted protein sequences. Gaps in the sequence may represent incomplete sequencing of mRNA (eg Alpaca, Duck where the 5’ sequence and AUG start codon has not been identified) or real evolutionary differences (eg the non-mammalian species which appear to lack the additional exons of the longer form seen in mammals). Colour coding as for Figure 2. **B.** Percent identity matrix calculated by Clustal Omega.

**A.**

kangaroo_rat MALPACAARALGPPPPPPLQPEQGAPARTTCPRRHSRAAAELAASLSGSVAASVRVGPPR 60

chinese_hamster MALPAFAARTLGP----PLQPEQGAPARTTCPRRHSRVEAELAASRPGSVAASVHASPPR 56

rat MALPAFAARALGP----PLQPEQGAPARTTCPRRHSRVEAELAASRPGSVAASVRAGPPR 56

mouse_reference_C57BL MALPAFAARALGP----PLQPEQGAPARTTCPRRHSRVEAELAASRPGSVAASVRAGPPR 56

mouse_BALBc MALPAFAARALGP----PLQPEQGAPARTTCPRRHSRVEAELAASRPGSVAASVRAGPPR 56

guinea_pig MALPACAARALGP----PLQPEHGAPARTTCPRRHSRVEAELAASRPGSVSASVRAGPPR 56

human MALPACAVREFEP----PRQPERGAPVRTTCPRRHSRVEAELAASRPGSVAASVRAGPPR 56

alpaca ------------------------------------------------------------ 0

horse MALPACAAPALRP----PLQPERGAPARTTCPRRHSRVEAELAASRPGSVAASVRAGPPR 56

dog MALPACAAQALGP----PLQPERGAPARTTCPRRHSRVEAELAGSRPGSVAASVRAGPPR 56

cat MALPACAARALGP----PLQPERRAPARTTCPRRHSRVEAELAASRPGSVAASVLAGPPR 56

xenopus ------------------------------------------------------------ 0

flycatcher ------------------------------------------------------------ 0

duck ------------------------------------------------------------ 0

chicken ------------------------------------------------------------ 0

turkey ------------------------------------------------------------ 0

opossum ------------------------------------------------------------ 0

wallaby ------------------------------------------------------------ 0

cod ------------------------------------------------------------ 0

stickleback ------------------------------------------------------------ 0

tetraodon ------------------------------------------------------------ 0

zebrafish ------------------------------------------------------------ 0

cave_fish ------------------------------------------------------------ 0

kangaroo_rat GVSHRFNSQPLQDESLKACSSLAGAVRAPLFALLPRGRRRRMHDFRRRWDLGSLCRALLT 120

chinese_hamster GVSLGFNSPPLQDKPPKAFSSLAGALRAPLFALLPRGRRRRMHDLRRRWDLGSLCRALLT 116

rat GVSLGFNSPPLQDKPPKTFSSLAGALRAPLFALLPRGRRRRMHDLRRRWDLGSLCRALLT 116

mouse_reference_C57BL GVSLGFNSPPLQDKPPKAFSSLAGALRAPLFALLPRGRRRRMHDLRRRWDLGSLCRALLT 116

mouse_BALBc GVSLGFNSPPLQDKPPKAFSSLAGALRAPLFALLPRGRRRRMHDLRRRWDLGSLCRALLT 116

guinea_pig GVSHGFNSRQLLDEPLKASASLAGALRAPLFALLPRGRRRRMNDLRRRWDLGSLCRALLT 116

human GVSHGFHTRPLLDKPRKASSSLAGAACAPLFALLSRGRRRRMHVLRRRWDLGSLCRALLT 116

alpaca ------------------------------------------------------------ 0

horse GVSRGFNSQPLLDEPLKASSSLAGAARAPLFALLPRGRRRRMHDLRRRWDLGSLCRALLT 116

dog GVSRGFNSQPLLDEPLKASSSPAGAARTPLFALLPRGRRRRMHDLRRRWDLGSLCRALLT 116

cat GVSRGFNSQPLLDEPPKASSSLAGAARTSLFALLPRSRRRRMHDLRRRWDLGSLCRALLT 116

xenopus ------------------------------------------------------------ 0

flycatcher ------------------------------------------------------------ 0

duck ------------------------------------------------------------ 0

chicken ------------------------------------------------------------ 0

turkey ------------------------------------------------------------ 0

opossum ------------------------------------------------------------ 0

wallaby ------------------------------------------------------------ 0

cod ------------------------------------------------------------ 0

stickleback ------------------------------------------------------------ 0

tetraodon ------------------------------------------------------------ 0

zebrafish ------------------------------------------------------------ 0

cave_fish ------------------------------------------------------------ 0

kangaroo_rat RGLAALGHSLKHVLSAIFSKIFGPLASVGNMDEKSNKLLLALVMLFLFAVIVLQYVCPG- 179

chinese_hamster RGLAAVGHSLKHVLSAIFSKIFGPLASVGNMDEKSNKLLLALVMLFLFAVIVLQYVCPG- 175

rat RGLAAVGHSLKHVLSAIFSKIFGPLASVGNMDEKSNKLLLALVMLFLFAVIVLQYVCPG- 175

mouse_reference_C57BL RGLAAVGHSLKHVLSAIFSKIFGPLASVGNMDEKSNKLLLALVMLFLFAVIVLQYVCPG- 175

mouse_BALBc RGLAAVGHSLKHVLSAIFSKIFGPLASVGNMDEKSNKLLLALVMLFLFAVIVLQYVCPG- 175

guinea_pig RGLAALGHSLKHVLSAIFSKIFGPLASVGNMDEKSNKLLLALVMFFLFAVIVLQYVCPG- 175

human RGLAALGHSLKHVLGAIFSKIFGPMASVGNMDEKSNKLLLALVMLFLFAVIVLQYVCPG- 175

alpaca ---------------------------VGNMDEKSNKLLLALVMLFLFAVIVLQYVCPG- 32

horse RGLAALGHSLKHVLGAIFSKIFGPLASVGNMDEKSNKLLLALVMLFLFAVIVLQYVCPG- 175

dog RGLAALGHSLKHVLGAIFSKIFGPLASVGNMDEKSNKLLLALVMLFLFAVIVLQYVCPG- 175

cat RGLAALGHSLKHVLGAIFSKIFGPLASVGNMDEKSNKLLLALVMLFLFAVIVLQYVCPG- 175

xenopus ------------------------------------------------------------ 0

flycatcher ------------------------------MEDRSHKVLLALVMLFLFAVIVLQYVCPG- 29

duck ------------------------------------------------------------ 0

chicken ------------------------------MEDRSHKVLLALVMLFLFAVIVLQYVCPG- 29

turkey ---------------------------------RSHKVLLALVMLFLFAVIVLQYVCPG- 26

opossum ------------------------------MDDKSNKLLLALVMLFLFAVIVVQYVCPG- 29

wallaby -----------------------------TMDDKSNKLLLALVMLFLFAVIVLQYVCPG- 30

cod ---------------------------MDEKSSHSHRLLIVLLMLMLFGVIVLQYVCPGT 33

stickleback --------------------------MDDKSSSSHHRLLVVLLMVLLFGVIMIQYVCPSK 34

tetraodon -----------------------------GGQGPQQRLLAVLVLVLLFGVMLVQYICPGR 31

zebrafish ---------------------------MDG-KSNYSRLLIALLMILFFGGIVLQYICST- 31

cave_fish ---------------------------MDE-KSGSSRLLVALVTVLLFGAIVLQYVCPG- 31

kangaroo_rat TECQLLRLQAF--------------------SS-PVPDPYRSEDESSARFVPRYNFSRGD 218

chinese_hamster TECQLLRLQAF--------------------SS-PVPDPYRSEDESSARFVPRYNFSRSD 214

rat TECQLLRLQAF--------------------SS-PVPDPYRSEDESSARFVPRYNFSRGD 214

mouse_reference_C57BL TECQLLRLQAF--------------------SS-PVPDPYRSEDESSARFVPRYNFSRGD 214

mouse_BALBc TECQLLRLQAF--------------------SS-PVPDPYRSEDESSARFVPRYNFSRGD 214

guinea_pig TECQLLRLQAF--------------------SS-PLPDPYRSEDESSARFVPRYNFSRGD 214

human TECQLLRLQAF--------------------SS-PVPDPYRSEDESSARFVPRYNFTRGD 214

alpaca TECQLLRLQAF--------------------SS-PMPDPYRSEDESSARFVPRYNFSRGD 71

horse TECQLLRLQAF--------------------SS-PMPDPYRSEDESSARFVPRYNFSRGD 214

dog TECQLLRLQAF--------------------SS-PMPDPYRSEDESSSRFVPRYNFSRGD 214

cat TECQLLRLQAF--------------------SS-PMPDPYRSEDESSARFVPRYNFSRGD 214

xenopus ------------------------------------------------------------ 0

flycatcher TECQLLRLRAL--------------------SPAAAADPYRAEDETPARFVPRFNFSADD 69

duck ------------------------------------------------------------ 0

chicken TECQLLRLRAL--------------------SPAAAADPYRAEDETPARFVPRFNFSAGD 69

turkey TECQLLRLRAL--------------------SPAAAADPYRAEDETPARFVPRFNFSAGD 66

opossum TECQLLRLQAF--------------------AS-SHADPYRTEDESPPRFVARLNFTDAE 68

wallaby TECQLLRLQAF--------------------AS-P-MLPYRTEDESPPRFVARLNFTDAE 68

cod SDCQILR-IGSIFKSNSASDA---PKGD--GKDGSPKDPYISEDNALVRFVPRFNFTKAD 87

stickleback SECQMLHQLGTWFKDGAATGS---PRGGDQIQDGLQKDPYIAEDGALVRFVPRFNFTDVD 91

tetraodon SECPVLHQLGSWFREP-----------GRETQDSLLRDPYIAEDGALVRFVPRFNFSKAD 80

zebrafish SDWQLLHLASLSSRLG--S-----RAPGDRLNGAGAGDPYSSEDGALVRFVPRFNFTTKD 84

cave_fish SDCQLLRLGSSGAAGDGGSGAGGGGGGSGASGAAGAGDPYVAEDGALARFVPRFHFSAAD 91

kangaroo_rat LLRKVDFDIKGDDLIVFLHIQKTGGTTFGRHLVRNIQLEQPCECRVGQKKCTCHRPGKRE 278

chinese_hamster LLRKVDFDIKGDDLIVFLHIQKTGGTTFGRHLVRNIQLEQPCECRVGQKKCTCHRPGKRE 274

rat LLRKVDFDIKGDDLIVFLHIQKTGGTTFGRHLVRNIQLEQPCECRVGQKKCTCHRPGKRE 274

mouse_reference_C57BL LLRKVDFDIKGDDLIVFLHIQKTGGTTFGRHLVRNIQLEQPCECRVGQKKCTCHRPGKRE 274

mouse_BALBc LLRKVDFDIKGDDLIVFLHIQKTGGTTFGRHLVRNIQLEQPCECRVGQKKCTCHRPGKRE 274

guinea_pig LLRKVDFDINGDDLIVFLHIQKTGGTTFGRHLVRNIQLEQPCECRVGQKKCTCHRPGKRE 274

human LLRKVDFDIKGDDLIVFLHIQKTGGTTFGRHLVRNIQLEQPCECRVGQKKCTCHRPGKRE 274

alpaca LLRKVDFDIKGDDLIVFLHIQKTGGTTFGRHLVRNIQLEQPCECRVGQKKCTCHRPGKRE 131

horse LLRKVDFDIKGDDLIVFLHIQKTGGTTFGRHLVRNIQLEQPCECRVGQKKCTCHRPGKRE 274

dog LLRKVDFDIKGDDLIVFLHIQKTGGTTFGRHLVRNIQLEQPCECRVGQKKCTCHRPGKRE 274

cat LLRKVDFDIKGDDLIVFLHIQKTGGTTFGRHLVRNIQLEQPCECRVGQKKCTCHRPGKRE 274

xenopus ------------------------------------------------------------ 0

flycatcher LLRRVDFNIKGDDLIVFLHIQKTGGTTFGRHLVRNIQLEQPCECRAGQKKCTCHRPGKRE 129

duck ------------------------------------------------------------ 0

chicken LLRRVDFNIKGDDLIVFLHIQKTGGTTFGRHLVRNIQLEQPCECRAGQKKCTCHRPGKRE 129

turkey LLRRVDFNIKGDDLIVFLHIQKTGGTTFGRHLVRNIQLEQPCECRAGQKKCTCHRPGKRE 126

opossum LLRKVDFNIKGDDLIVFLHIQKTGGTTFGRHLVRNIQLEQPCECKAGQKKCTCHRPGKRE 128

wallaby LLRKVDFNIKGDDLIVFLHIQKTGGTTFGRHLVRNIQLEQPCECKAGQKKCTCHRPGKRE 128

cod LSRAVDFNIKGDDVIVFLHIQKTGGTTFGRHLVRNIQLERPCECHAGQKKCTCLRPGKKE 147

stickleback LNRAVDFNIKGDDVIVFLHIQKTGGTTFGRHLVRNIQLERPCECHAGQKKCTCFRPGKKE 151

tetraodon LNRVVDFNINGDDVIVFLHIQKTGGTTFGRHLVRNIKLERPCDCRAGQKKCTCYRPGKKE 140

zebrafish LSRAVDFHIKGDDVIVFLHIQKTGGTTFGRHLVRNIQLERPCECHAGQKKCTCYRPGKRD 144

cave_fish LDRVVDFNIKGDDVIVFLHIQKTGGTTFGRHLVRNIQLERPCECRAGQKKCTCYRPGKRE 151

kangaroo_rat TWLFSRFSTGWSCGLHADWTELTSCVPAVVDGKRDARLRPS------------------- 319

chinese_hamster TWLFSRFSTGWSCGLHADWTELTSCVPAVVDGKRDARLRPSRWRIFQILDSTSKDRWGSS 334

rat TWLFSRFSTGWSCGLHADWTELTSCVPAVVDGKRDARLRPSRWRIFQILDAASKDRWGSS 334

mouse_reference_C57BL TWLFSRFSTGWSCGLHADWTELTSCVPAVVDGKRDARLRPSRWRIFQILDGTSKDRWGSS 334

mouse_BALBc TWLFSRFSTGWSCGLHADWTELTSCVPAVVDGKRDARLRPSRWRIFQILDGTSKDRWGSS 334

guinea_pig TWLFSRFSTGWSCGLHADWTELTSCVPAVVDGKRDARLRPSRWRIFQILDAASKDRRGSP 334

human TWLFSRFSTGWSCGLHADWTELTSCVPSVVDGKRDARLRPSRWRIFQILDAASKDKRGSP 334

alpaca TWLFSRFSTGWSCGLHADWTELTSCVPAVVDGKRDARLRPSRWRIFQILDAASKDKRGSA 191

horse TWLFSRFSTGWSCGLHADWTELTSCVPAVVDGKRDARLRPSRWRIFQILDASSKDRRDSP 334

dog TWLFSRFSTGWSCGLHADWTELTSCVPAVVDGKRDARLRPSRWRIFQILDAASKDRRGSP 334

cat TWLFSRFSTGWSCGLHADWTELTSCVPAVVDGKRDARLRPSRWRIFQILDAASKDRRGSP 334

xenopus ---------------------------PPHPEVPHNHLHT-------------------- 13

flycatcher TWLFSRFSTGWSCGLHADWTELTNCVPSVVDSKKEVRLRP-------------------- 169

duck ------------------------------------------------------------ 0

chicken TWLFSRFSTGWSCGLHADWTELTNCVPSVVDSKKEVRLRP-------------------- 169

turkey TWLFSRFSTGWSCGLHADWTELTNCVPSVVDSKKEVRLRP-------------------- 166

opossum TWLFSRFSTGWSCGLHADWTELTNCVPSIVDSKKEAKLRPTSCCKSKKAHCVCRD----- 183

wallaby TWLFSRFSTGWSCGLHADWTELTNCVPSIVDSKKEVKLRPT------------------- 188

cod TWLFSRFSTGWSCGLHADWTELTNCVPTRMDTHEAP------------------------ 183

stickleback TWLFSRFSTGWSCGLHADWTELTSCVPSRMDSREAP------------------------ 187

tetraodon TWLFSRFSTGWSCGLHADWTELTSCVPSRMDSVEVL------------------------ 176

zebrafish TWLFSRFSTGWSCGLHADWTELTNCVPSFMSNRESQE-R--------------------- 182

cave_fish TWLFSRFSTGWSCGLHADWTELTNCVPAVMGNREPVETH--------------------- 190

kangaroo_rat ---------------------RNFHYITILRDPVSRYLSEWRHVQRGATWKASLHVCDGR 358

chinese_hamster NFNSGANSPSSTKTRSTSKSGKNFHYITILRDPVSRYLSEWRHVQRGATWKASLHVCDGR 394

rat NFNSGANSPSSTKPRSTTKSGKNFHYITILRDPVSRYLSEWRHVQRGATWKASLHVCDGR 394

mouse_reference_C57BL NFNSGANSPSSTKPRSTSKSGKNFHYITILRDPVSRYLSEWRHVQRGATWKASLHVCDGR 394

mouse_BALBc NFNSGANSPSSTKPRSTSKSGKNFHYITILRDPVSRYLSEWRHVQRGATWKASLHVCDGR 394

guinea_pig NTNPGANSPS-TKTRNTSKSGKNFHYITILRDPVSRYLSEWRHVQRGATWKASLHVCDGR 393

human NTNAGANSPSSTKTRNTSKSGKNFHYITILRDPVSRYLSEWRHVQRGATWKASLHVCDGR 394

alpaca NINPGANSPSSTKARNTSKNGKNFHYITILRDPVSRYLSEWRHVQRGATWKASLHVCDGR 251

horse KTNPGATSPASTKARNTSKSGKNFHYITILRDPVSRYLSEWRHVQRGATWKASLHVCDGR 394

dog NTNPGANSPSSTKARNTSKSGKNFHYITILRDPVSRYLSEWRHVQRGATWKASLHVCDGR 394

cat NTNPGANSPSSTKARNASKSGKNFHYITILRDPVSRYLSEWRHVQRGATWKASLHVCDGR 394

xenopus --------------------SRNFYYITILRDPVSRYLSEWRHVQRGATWKASLHVCDGR 53

flycatcher --------------------SRNFYYITILRDPVSRYLSEWRHVQRGATWKASLHVCDGR 209

duck ---------------------RNFYYITILRDPVSRYLSEWRHVQRGATWKASLHVCDGR 39

chicken --------------------SRNFYYITILRDPVSRYLSEWRHVQRGATWKASLHVCDGR 209

turkey --------------------SRNFYYITILRDPVSRYLSEWRHVQRGATWKASLHVCDGR 206

opossum --------PKGAYQRRGTKKRRNFYYITILRDPVSRYLSEWRHVQRGATWKASLHVCDGR 235

wallaby ----------------------NFYYITILRDPVSRYLSEWRHVQRGATWKASLHVCDGR 248

cod ---------------------RNYYYITILRDPVWRYLSEWRHVQRGATWKASLHVCDGH 222

stickleback ----------------VDLPSRNYYYITILRDPVSRYLSEWRHVQRGATWRASLHVCDGR 231

tetraodon ----------------ENLSSRNYYYITILRDPVSRYLSEWRHVQRGATWKASLHVCDGR 220

zebrafish ----------------RMTPSRNYYYITILRDPVWRYLSEWRHVQRGATWKASKHMCDGR 226

cave_fish ----------------RTAPSRNYYYITILRDPVWRYLSEWRHVQRGATWKASLHMCDGR 234

*::********* ***************:** *:***:

kangaroo_rat PPTSEELPSCYTGDDWSGCPLKEFMDCPYNLANNRQVRMLSDLTLVGCYNLSVMPEKQRN 418

chinese_hamster PPTSEELPSCYTGDDWSGCPLKEFMDCPYNLANNRQVRMLSDLTLVGCYNLSVMPEKQRN 454

rat PPTSEELPSCYTGDDWSGCPLKEFMDCPYNLANNRQVRMLSDLTLVGCYNLSVMPEKQRN 454

mouse_reference_C57BL PPTSEELPSCYTGDDWSGCPLKEFMDCPYNLANNRQVRMLSDLTLVGCYNLSVMPEKQRN 454

mouse_BALBc PPTSEELPSCYTGDDWSGCPLKEFMDCPYNLANNRQVRMLSDLTLVGCYNLSVMPEKQRN 454

guinea_pig PPTSEELPSCYTGDDWSGCPLKEFMDCPYNLANNRQVRMLSDLTLVGCYNLSVMPEKQRN 453

human PPTSEELPSCYTGDDWSGCPLKEFMDCPYNLANNRQVRMLSDLTLVGCYNLSVMPEKQRN 454

alpaca PPTSEELPSCYTGDDWSGCPLKEFMDCPYNLANNRQVRMLSDLTLVGCYNLSVMPEKQRN 311

horse PPTSEELPSCYTGDDWSGCPLKEFMDCPYNLANNRQVRMLSDLTLVGCYNLSVMPEKQRN 454

dog PPTSEELPSCYTGDDWSGCPLKEFMDCPYNLANNRQVRMLSDLTLVGCYNLSVMPEKQRN 454

cat PPTSEELPSCYTGDDWSGCPLKEFMDCPYNLANNRQVRMLSDLTLVGCYNLSVMPEKQRN 454

xenopus SPTSEELPSCYTGDDWSGCSLKEFMDCPYNLANNRQVRMLSDLILVGCYNLSVMPEDQRN 113

flycatcher SPTTEELPSCYSGDDWSGCSLQEFMDCPYNLANNRQVRMLSDLSLVGCYNLSVMPEEQRN 269

duck SPTTEELPSCYTGDDWSGCSLQEFMDCPYNLANNRQVRMLSDLSLVGCYNLSVMPEEQRN 99

chicken SPTTEELPSCYTGDDWSGCSLQEFMDCPYNLANNRQVRMLSDLSLVGCYNLSVMPEEQRN 269

turkey SPTTEELPSCYTGDDWSGCSLQEFMDCPYNLANNRQVRMLSDLSLVGCYNLSVMPEEQRN 266

opossum SPTIEELPSCYAGDDWSGCSLKEFMDCPYNLANNRQVRMLSDLSLVGCYNLSVMPEEQRN 295

wallaby SPTIEELPSCYTGDDWSGCSLKEFMDCPYNLANNRQVRMLSDLSLVGCYNLSVMPEEQRN 308

cod SPTLSELPSCYLGDDWSGCTLQEFMDCPYNLANNRQTRMLADLSLVGCYNVSAMGDEDRS 282

stickleback SPTLSELPSCYPGDDWSGCSLQEFMDCPYNLANNRQTRMLADLSLVGCYNVSTMNEDERW 291

tetraodon SPTLSELPSCYSGDDWSGCSLQEFMDCAYNLANNRQTRMLADLSLVGCYNVSAMSEEARW 280

zebrafish LPTLTELPSCYPGDDWSGCSLEEFMVCPYNLANNRQTRMLADLSLVGCYNLTVMSENQRW 286

cave_fish APTVAELPSCYQGDDWSGCSLEEFMACPHNLANNRQTRMLADLSLVGCYNLTAMSDSQRG 294

** ****** ******* *:*** * :*******.***:** ******::.* :. *

kangaroo_rat KVLLESAKSNLKHMAFFGLTEFQRKTQYLFEKTFNMNFISPFTQYNTTRASSVEINGEIQ 478

chinese_hamster KVLLESAKSNLKHMAFFGLTEFQRKTQYLFEKTFNMNFISPFTQYNTTRASSVEINEEIQ 514

rat KVLLESAKSNLKHMAFFGLTEFQRKTQYLFEKTFNMNFISPFTQYNTTRASSVEINEEIQ 514

mouse_reference_C57BL KVLLESAKSNLKHMAFFGLTEFQRKTQYLFEKTFNMNFISPFTQYNTTRASSVEINEEIQ 514

mouse_BALBc KVLLESAKSNLKHMAFFGLTEFQRKTQYLFEKTFNMNFISPFTQYNTTRASSVEINEEIQ 514

guinea_pig KVLLESAKSNLKHMAFFGLTEFQRKTQYLFEKTFNMNFISPFTQYNTTRASSVEINEDIQ 513

human KVLLESAKSNLKHMAFFGLTEFQRKTQYLFEKTFNMNFISPFTQYNTTRASSVEINEEIQ 514

alpaca KVLLESAKSNLKHMAFFGLTEFQRKTQYLFEKTFNMNFISPFTQYNTTRASSVEINEEIQ 371

horse KVLLESAKTNLKHMAFFGLTEFQRKTQYLFEKTFNMNFISPFTQYNTTRASSVEINEEIQ 514

dog KVLLESAKSNLKHMAFFGLTEFQRKTQYLFEKTFNMNFISPFTQYNTTRASSVEINEEIQ 514

cat KVLLESAKSNLKHMAFFGLTEFQRKTQYLFEKTFNMNFISPFTQYNTTRASSVEINEEIQ 514

xenopus KVLLDSAKENLKRMAFFGLTEFQRKTQYLFEKTFNMNFISPFTQFNSTRASSVEIDEQTQ 173

flycatcher KVLLDSAKENLKRMAFFGLTEFQRKTQYLFEKTFNMNFISPFTQYNSTRASSVEIDRQTQ 329

duck KVLLDSAKENLKRMAFFGLTEFQRKTQYLFEKTFNMNFISPFTQYNSTRASSVEIDEQTQ 159

chicken KVLLDSAKENLKRMAFFGLTEFQRKTQYLFEKTFNMNFISPFTQYNSTRASSVEIDEQTQ 329

turkey KVLLDSAKENLKRMAFFGLTEFQRKTQYLFEKTFNMNFISPFTQYNSTRASSVEIDEQTQ 326

opossum KVLLDSAKENLKCMAFFGLTEFQRKTQYLFEKTFNMNFISPFTQYNNTRASSVEIDQQTQ 355

wallaby KVLLDSAKENLKCMAFFGLTEFQRKTQYLFEKTFNMNFISPFTQYNNTRASSVEIDQQTQ 368

cod AVLLESAKRNLRGMAFFGLTEYQRKTQYLFERTFRLAFIAPFTQLNGTRAASVEVALDTQ 342

stickleback ALLVESAKRNLRGMAFFGLTEYQRKTQYLFERTFNLEFIAPFTQLNGTRASGVDVPDETQ 351

tetraodon AVLLESAKRNLRSMAFFGLTEYQRKTQYLFERTFHLEFITPFTQVNGTRASSVEVPPKTQ 340

zebrafish AMLLESAKRNLRNMAFFGLTEYQRKTQYLFEHTFRLSFIAPFTQLNGTRAASVEVEPETQ 346

cave_fish AVLLESAKRNLRRMAFFGLTEYQRKTQYLFERTFRLAFIAPFTQLNGTRAASVEVATETQ 354

:*::*** **: ********:*********:**.: **:**** * ***:.*:: . *

kangaroo_rat KLIEGLNFLDMELYSYAKDLFLQRYQFMRQKEHQEARRKRQEQRKFLKGRFLQTH-FQSQ 537

chinese_hamster KRIEGLNFLDMELYSFAKDLFLQRYQFMRQKEHQDARRKRQEQRKFLKGRFLQTH-FLSQ 573

rat KRIEGLNFLDMELYSYAKDLFLQRYQFMRQKEHQDARRKRQEQRKFLKGRFLQTH-FQSQ 573

mouse_reference_C57BL KRIEGLNFLDMELYSYAKDLFLQRYQFMRQKEHQDARRKRQEQRKFLKGRFLQTH-FQSQ 573

mouse_BALBc KRIEGLNFLDMELYSYAKDLFLQRYQFMRQKEHQDARRKRQEQRKFLKGRFLQTH-FQSQ 573

guinea_pig KRIERLNFLDMELYSYAKDLFLQRYQFMRQKEHQEARRKRQEQRKFLKGRFLQTH-FQSQ 572

human KRIEGLNFLDMELYSYAKDLFLQRYQFMRQKEHQEARRKRQEQRKFLKGRLLQTH-FQSQ 573

alpaca KRIEGLNFLDMELYSYAKDLFLQRYQFMRQKEHQEARRKRQEQRKFLKGRFLQTH-FQSQ 430

horse KRIEGLNFLDMELYSYAKDLFLQRYQFMRQKEHQEARRKRQEQRKFLKGKLLQTH-FQSQ 573

dog KRIEGLNFLDMELYSYAKDLFLQRYQFMRQKEHQEARQKRQEQRKFLKGRFLQTH-FQSQ 573

cat KRIEGLNFLDMELYSYAKDLFLQRYQFMRQKEHQEARQKRQEQRKFLKGRFLQTH-FQSQ 573

xenopus KRIEALNFLDMELYEYAKDLFLQRYQFMRQKEHQEARRKRQEQRKILRAKHLIHK-EE-- 230

flycatcher QRIEALNFLDVELYEYAKDLFLQRYQYMRQKEHQEARRKRQEQRKILRAQQARLR-EQG- 387

duck QRIEALNFLDMELYDYAKDLFLQRYQYMRQKEHQEARRKRQEQRKILRAKQAHLK-EQG- 217

chicken QRIEALNFLDMELYDYAKDLFLQRYQYMRQKEHQEARRKRQEQRKILRAKQAHLR-EQG- 387

turkey QRIEALNFLDVELYDYAKDLFLQRYQYMRQKEHQEARRKRQEQRKILRAKQAHLR-EQG- 384

opossum KRIEALNFLDMELYEYAKDLFLQRYQFTRQKDHQEARRKRQE-RKILKTRHVHKR-DQ-- 411

wallaby KRIEALNFLDMELYEYAKDLFLQRYQFTRQKDHQEARRKRQEQRKILKTRHAHNR-DQ-- 425

cod RRIRGLNRWDVELYEYARDLFLQRFQRARQQERRQARERRQQERR--RLRGRLSSARPG- 399

stickleback RRILRLNRWDLELYEYARDLFLQRFQVARQQERRRARARRQQERRRRLLRGRLV-AKPG- 409

tetraodon QRILQLNHWDLELYEYARDLFLQRFQVARQQERRQARERRQQERR--RLRGRFT-AKQR- 396

zebrafish RRIRELNQWDVELYEYARDLFLQRFQFARQQERREARQRRIQERRKLRAKVKSWLGVTGK 406

cave_fish QRIRELNRWDVELYEYARDLFLQRFQYARQQERRKARQRRQQERRRLRAKVLPWWGGPGK 414

: * ** *:***.:*:******:* **:::: ** :* : *: :

kangaroo_rat GQ----------GQSRNPGQNQNQNPNQN---NVTHNLMQNLTQS----SSQKENRVRQK 580

chinese_hamster SQSQS----QSQGQNQSPGQNLSQSPNPNPNQNLTQNVSQNLTQSSKPNLIRKENRGSQK 629

rat SQSQGQSQGQSQGQSQGPGQNLSQNPNPNPNQNLTQNLSQNLTQSSNPNLTQRENHGSQK 633

mouse_reference_C57BL SQGQ--------SQSQSPGQNLSQNPNPNPNQNLTQNLSHNLTPSSNPNSTQRENRGSQK 625

mouse_BALBc SQGQ--------SQSQSPGQNLSQNPNPNPNQNLTQNLSHNLTPSSNPNSTQRENRGSQK 625

guinea_pig SQ----------SQSQNPSQNQSQNPNPNANQSLTQNLIQNLTQNSSQNLSQKENRSNQK 622

human GQ----------GQSQNPNQNQSQNPNPNANQNLTQNLMQNLTQSL----SQKENRESPK 619

alpaca SQ----------GQNQNLSQNPSQNPNPNVNQNVTQNVTQNLTQSSGQNSSLKENQESQK 480

horse SQ----------GQSQNLSQNQSQNPNLNANQNVTQNLIQNLTQTSSQNSSQKENRESQR 623

dog GQ----------GQSQNPSQNQSQNPNLNVNQNVTQNLIQNLTQ----NLSHKENRESQK 619

cat G------------QSQNPSQNPSQNPNLNANQNVTQNLIQNLTQNSSQNSSHKENRESQK 621

xenopus ------------------------------------------------------------ 230

flycatcher ------------------------------------------------------------ 387

duck ------------------------------------------------------------ 217

chicken ------------------------------------------------------------ 387

turkey ------------------------------------------------------------ 384

opossum ------------------------------------------------------------ 411

wallaby ------------------------------------------------------------ 425

cod RPLRPTEEP------PAPGR---PPG------------------------A--SRPRRGE 424

stickleback RQLKPTDAP------RPPPS---RSV------------------------VAEEEQLRGK 436

tetraodon GQQKPTETP------RHPDR---QRA------------------------ASQE------ 417

zebrafish AVFKPTKEP------PMTE----QSPA-----------------------FAEEKQADAE 433

cave_fish GPKP-TKQS------PVRQS---VSTR-----------------------PAEPKQVE-- 439

kangaroo_rat QSPGQEQ---------SDN--PTNGTHDYIGSVEKWR- 606

chinese_hamster QGSGQEQ---------SHSS-TSNGTNDYIGSVETWR- 656

rat QGSGQEQ---------SDSS-TSNGTNDYIGSVETWR- 660

mouse_reference_C57BL QGSGQGQ---------GDSG-TSNGTNDYIGSVETWR- 652

mouse_BALBc QGSGQGQ---------GDSG-TSNGTNDYIGSVETWR- 652

guinea_pig QSPGHKQ---------NDSSSGSSGTNDYIGSVEKWR- 650

human QNSGKEQ---------N-DN-TSNGTNDYIGSVEKWR- 645

alpaca QSPGQEP---------GDGH-TSPGTSDYMGSVEKWR- 507

horse QNPGQEQ---------SDSN-PSNGTNDYIGSVEKWR- 650

dog QNPGQEQ---------SDGN-TSNGTNDYIGSVEKWR- 646

cat QSAGQEQ---------SDGN-TSNGTNDYIGSVEKWR- 648

xenopus ---------------------TDNSTNDYIGNVEKW-- 245

flycatcher ---------------------ENSSSADYTGNVERWRR 404

duck ---------------------ENSSSTDYIGNVERWR- 233

chicken ---------------------ENSSSTDYLGNVERWR- 403

turkey ---------------------ENSSSTDYLGNVERWR- 400

opossum ---------------------TDNATADYVEDVEKWR- 427

wallaby ---------------------TDNATADYGEDVEQWR- 441

cod GGGETLEAEGLLPDWWDLE--ENGTLEDYTDNVEQWW- 459

stickleback AGAGGEASEVLLPDWWDLE--ENGTMEDYMDNVEQWK- 471

tetraodon NAGDDTQAEILLPDLWDQD--ENATVDDYMDNVEQW-- 451

zebrafish RTLESETEGQVEENWLEED--DGEIMLDYLENVEQWR- 468

cave_fish TEKNQENEGELEDLWWEEENDENDTIEDYTENVEQW-- 475

** .** *

**B.**
